# Androgen Signaling in Uterine Diseases: New Insights and New Targets

**DOI:** 10.3390/biom12111624

**Published:** 2022-11-03

**Authors:** Mu Lv, Juanjuan Yu, Yan Huang, Jie Ma, Jun Xiang, Yanqiu Wang, Linxia Li, Zhenbo Zhang, Hong Liao

**Affiliations:** 1 Department of Obstetrics and Gynecology, Shanghai General Hospital, Shanghai Jiao Tong University School of Medicine, Shanghai 200080, China; 2 Department of Gynecologic Oncology, Fudan University Shanghai Cancer Center, 270 Dong-An Road, Shanghai 200032, China; 3 Department of Urology, Tongji Hospital, School of Medicine, Tongji University, Shanghai 200065, China; 4 Reproductive Medicine Center, Department of Obstetrics and Gynecology, Tongji Hospital, School of Medicine, Tongji University, Shanghai 200065, China; 5 Department of Obstetrics and Gynecology, Seventh People’s Hospital of Shanghai University of Traditional Chinese Medicine, 358 Datong Road, Shanghai 200137, China; 6 Shanghai Key Laboratory for Assisted Reproduction and Reproductive Genetics, Renji Hospital, School of Medicine, Shanghai Jiao Tong University, Shanghai 200135, China; 7 Department of Clinical Laboratory Medicine, Shanghai First Maternity and Infant Hospital, Tongji University School of Medicine, Shanghai 200040, China

**Keywords:** androgen, androgen receptor, uterine diseases, endometrium

## Abstract

Common uterine diseases include endometriosis, uterine fibroids, endometrial polyps, endometrial hyperplasia, endometrial cancer, and endometrial dysfunction causing infertility. Patients with uterine diseases often suffer from abdominal pain, menorrhagia, infertility and other symptoms, which seriously impair their health and disturb their lives. Androgens play important roles in the normal physiological functions of the uterus and pathological progress of uterine diseases. Androgens in women are synthesized in the ovaries and adrenal glands. The action of androgens in the uterus is mainly mediated by its ligand androgen receptor (AR) that regulates transcription of the target genes. However, much less is known about the signaling pathways through which androgen functions in uterine diseases, and contradictory findings have been reported. This review summarizes and discusses the progress of research on androgens and the involvement of AR in uterine diseases. Future studies should focus on developing new therapeutic strategies that precisely target specific AR and their related signaling pathways in uterine diseases.

## 1. Introduction

The uterus, as an important reproductive organ of women, is extremely sensitive to the actions of sex steroids. There are a variety of uterine diseases, including endometriosis, fibroids, endometrial polyps, endometrial hyperplasia (EH), endometrial cancer (EC), and infertility due to endometrial dysfunction. Androgens play an important role in the regulation of uterus function in health and disease. Dehydroepiandrosterone sulfate (DHEAS), dehydroepiandrosterone (DHEA), androstenedione (A4), testosterone (T), and dihydrotestosterone (DHT) are the most common androgens in women [1]. The first three kinds of androgens cannot play their role directly. They must be transformed into T and DHT in the target organ before binding to the androgen receptor (AR) [2]. There is growing evidence that androgens regulate important functions of the uterus, including endometrial proliferation, preparation for pregnancy, and tissue repair during menstruation. Dysregulation of androgen action is associated with endometrial diseases such as endometriosis and endometrial cancer and may cause infertility in women due to endometrial dysfunction [3]. Compared with the effects of progesterone and estrogen on the physiological and pathological uterus, limited information on the roles of androgens in uterus lesion is available, and contradictory findings have also been reported. This article discusses the recent research progress of androgens in uterine diseases and explores the potential of AR as a therapeutic target for treating uterine diseases.

## 2. Androgen and Androgen Receptor

### 2.1. Metabolism and Biological Activity of Androgen

**Figure 1:**
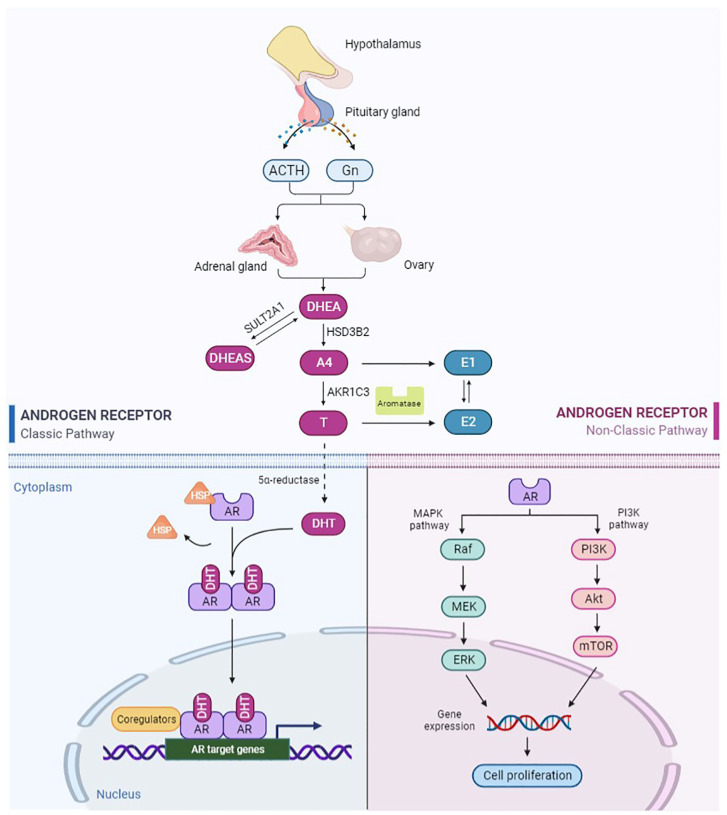
Schematic of androgen production, metabolism, and classical and nonclassical signaling pathways of AR. The figure was created with https://biorender.com (accessed on 1 September 2022).

In females, androgens are mainly produced by the ovaries and adrenal glands, which are controlled by gonadotropin (Gn) and adrenocorticotropic hormone (ACTH). DHEA and T are regarded as indicators of androgen secretion by the adrenal glands and ovaries, respectively. After menopause, estrogen production is markedly reduced, but the ovaries continue to produce androgens, including DHEA, A4, and T. In postmenopausal women, DHEA is the main source of androgens and estrogens. Approximately 20% of circulating DHEA is produced by the ovaries, and the remaining 80% comes from the adrenal glands [4]. DHEA is converted to DHEAS by the enzyme steroid sulfotransferase type 2A1 (SULT2A1), or to A4 by the enzyme 3β-hydroxysteroid dehydrogenase type 2 (HSD3B2) [5]. A4 is further converted to T under the action of 17β-hydroxysteroid dehydrogenase type 5 (17β-HSD type 5), also known as aldo-keto reductase family 1 member 3 (AKR1C3) [6]. Finally, T is converted to DHT by the action of 5α-reductase (Figure 1). The formed DHEAS, DHEA, A4, and T enter the systemic circulation, of which the latter three are mainly bound to sex hormone-binding globulin (SHBG). In women, 80% of androgens bind to SHBG, 19% bind to serum albumin, and only 1% are free. Free androgens are the only active androgens. The rest of the bound androgens are used as a reservoir of circulating androgens. Biologically active androgens, such as T and DHT, exert their effects primarily by binding to AR to regulate the expression of downstream target genes. In addition, androgens are converted by aromatase to estrogens, which act primarily through the estrogen receptor (ER). A4 and T are converted to estrone (E1) and estradiol (E2), respectively [7,8].

### 2.2. Structure and Function of AR

AR is a nuclear hormone receptor transcription factor [9]. AR in the uterus is mainly located in the endometrium, mesenchyme and myometrium. A higher level of AR expression has been reported in stromal cells than in epithelial cells [10]. Furthermore, AR expression varies with the menstrual cycle [11]. The structure of AR protein consists of three domains: transcriptional regulatory domain (N-terminal domain, NTD), DNA-binding domain (DBD), and ligand-binding domain (C-terminal domain, LBD) [12]. DBD is the most conserved region, and its main function is to bind DNA. LBD is the region that binds to AR to form a dimer, which is critical for AR activation. Androgens activate downstream target gene pathways mainly through the classical AR-binding pathway (Figure 1). First, androgens bind to AR. Then, AR is separated from heat shock proteins (HSP) and transferred to the nucleus. AR homodimers bind to the androgen response element (ARE) on the promoter region of the target gene and recruit coactivators, which finally regulate the transcription of target genes [13]. In addition, AR can be rapidly activated in the absence of ligands, known as the nonclassical pathway (Figure 1) [14]. This ligand-independent pathway may be related to AR phosphorylation or AR-related signaling factors [14]. This is a fast and short activation mode with a response time of only a few seconds to a few minutes. Noncanonical pathways are not involved in the regulation of transcription and translation of target genes. It includes the activation of the PI3K/AKT/mTOR (Phosphatidylinositide 3-kinases/Serine/threonine kinases of the AGC family/Mammalian target of rapamycin) signaling pathway, the MAPK/ERK (Mitogen-activated protein kinase/extracellularregulated protein kinase) signaling pathway, and these pathways interact with each other [15,16].

## 3. Androgen Signaling in Endometriosis

Endometriosis is a benign estrogen-dependent disease in which the ectopic endometrium implants in a location other than the endometrium [17]. The prevalence of endometriosis is 10% in women of childbearing age [18]. Severe endometriosis can result in extensive pelvic adhesions, which lead to pain and infertility. It has been reported that T levels in endometriosis lesions are 5–19 times higher than the corresponding serum levels [19]. Consistent with this finding, an increased concentration of T, but not DHT, was found in endometriosis lesions, suggesting that T is the predominant androgen in endometriosis [18]. In addition, Carneiro et al. found 5α-reductase in the cytoplasm of glandular and stromal cells of the ectopic endometrium. This enzyme may mediate the conversion of T to DHT to enhance AR stimulation [20]. These findings suggest that active androgens may be formed in endometriosis tissue and that both local and systemic androgens may act on ectopic endometrial cells. In addition, a study identified AR as an endometriosis-associated transcription factor, and 373 AR target genes were differentially expressed in endometriosis compared to normal endometrium [21]. What is more, AR gene polymorphism may be related to the pathogenesis of endometriosis. The AR gene has a polymorphic cytosine, adenine, and guanine (CAG) microsatellite in exon 1 that encodes a variable-length glutamine repeat in the amino-terminal domain of the AR protein [22]. Hsieh et al. found that individuals with the M genotype (21 CAG repeats) had a higher risk of developing endometriosis compared with individuals without the M genotype [23]. This suggests that the CAG repeat polymorphisms of AR may serve as useful markers to predict endometriosis.

The clinical therapy strategy of endometriosis is to remove or reduce ectopic endometrium. Treatments of endometriosis include surgical treatment and drug medication. Drug therapy mainly includes nonsteroidal anti-inflammatory drugs, progestins, androgens, oral contraceptives, and gonadotropin-releasing hormone (GnRH) analogues [24]. Androgen is regarded as an effective treatment for women who cannot tolerate oral contraceptives or high doses of progesterone. Danazol is a derivative of 17α-ethinyltestosterone that increases the serum level of free T [25]. Atrophy of the endometrium has been reported as a result of hyper-androgen status [26]. In addition, danazol decreases estrogen production by interfering with the secretion of follicle-stimulating hormone (FSH) and luteinizing hormone (LH) [26]. In this way, danazol produces a high androgenic and low estrogenic environment, thereby reducing the size of the lesions in endometriosis and relieving pain [27,28]. In 18 endometriosis patients implanted with an intrauterine device (IUD) containing 300–400 mg danazol, there were significant improvements in symptoms like dysmenorrhoea and dyspareunia [29]. Adenomyosis is a type of endometriosis characterized by the presence of ectopic endometrium in the myometrium and hyperplasia of adjacent smooth muscle [30]. Igarashi was the first to use an IUD containing 175 mg danazol for adenomyosis treatment. This treatment reduced the uterine size significantly, and 66.6% of patients became pregnant as a result [31]. A prospective study of women with adenomyosis found that after six months of danazol-loaded IUD treatment, 81% of the participants had complete remission of dysmenorrhea, and 76% of the participants had improved menstrual bleeding [32]. In a mouse model using an IUD containing danazol, the number of adenomyosis nodules decreased as the dose increased [33]. In terms of mechanism, danazol affects the proliferation of adenomyosis cells by inhibiting DNA synthesis and inducing apoptosis. In adenomyotic glands and stromal cells treated with danazol, ER and bcl-2 protein expression is decreased, and necrosis of apoptotic cells is increased [34]. Vaginal administration [35] or cervical injection of danazol [36] may also improve symptoms of pain, vaginal bleeding, and dyspareunia. However, the use of danazol is limited due to side effects such as liver dysfunction and weight gain.

## 4. Androgen Signaling in Uterine Fibroids

**Figure 2:**
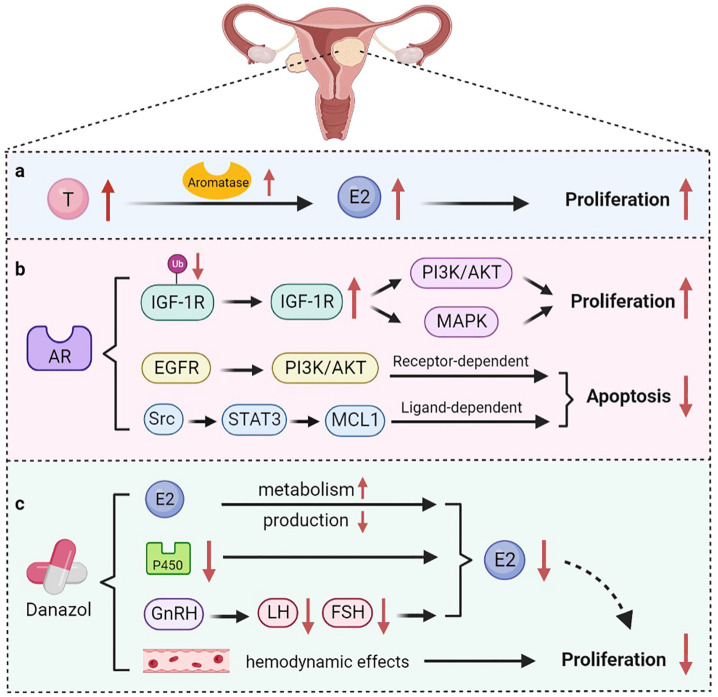
The role of androgen in uterine fibroids. (**a**) Testosterone is converted into E2 under the action of aromatase, thereby promoting the growth of uterine fibroids; (**b**) AR maintains the stability of IGF-1R protein and triggers the PI3K/AKT and MAPK pathways to promote cell proliferation. AR also mediates anti-apoptotic functions through receptor-dependent and ligand-dependent pathways; (**c**) Danazol normalizes estrogen metabolism and affects the production of FSH and LH, ultimately inhibiting estrogen production. Danazol can also affect the hemodynamic effects of uterine fibroids.

Uterine fibroids are benign tumors formed by the proliferation of smooth muscle tissue in the uterus. Depending on their location, uterine fibroids can be classified as intramural, submucosal, or subserosal types [37]. Uterine fibroids have a variety of clinical manifestations, including excessive menstruation, abdominal pain, anemia, and infertility, which affect the life quality of patients. High levels of estrogen in the body are a contributing factor to the development of uterine fibroids. Estrogen promotes the proliferation of uterine smooth muscle tissue by acting on ER [38]. Nevertheless, in vitro studies have shown that androgens are involved in the development of uterine fibroids as well (Figure 2) [39,40]. Wong et al. found that both high levels of T and E2 were associated with an increased risk of developing uterine fibroids [41]. Aromatase is overexpressed in uterine fibroids compared with normal myometrium, suggesting an interaction between androgen and estrogen in uterine fibroids [42]. The conversion of T to E2 might be the mechanism by which T promotes the growth of uterine fibroids [43].

AR was also found in uterine fibroids, suggesting a potential role for AR in the development of uterine fibroids [44]. Biopsies of 14 cases of fibroids and paired myometrial tissue revealed a 2–3-fold higher expression of AR and Ki-67 in fibroids than in normal myometrium [45]. AR exerts pro-proliferative and anti-apoptotic effects in myometrial cells through different signaling pathways (Figure 2). Insulin-like growth factor-1 (IGF-1) is an anabolic factor that regulates growth and differentiation. IGF-1 signaling has been reported to play a role in regulating proliferation of uterine leiomyomas [46]. AR inhibits the ubiquitination of the IGF-1 receptor (IGF-1R) protein, which, in turn, increases proliferation of myofibroblasts through the PI3K/AKT and MAPK pathways [47]. Furthermore, AR regulates the anti-apoptosis function of the downstream target gene MCL1 through receptor-dependent and ligand-dependent pathways [48]. In the receptor-dependent pathway, AR regulates the expression of MCL1 through the epidermal growth factor receptor (EGFR)/PI3K/AKT pathway. In the ligand-dependent pathway, AR triggers activation of Src kinase and the transcription factor STAT3, leading to increased expression of MCL1. These results suggest that AR is an important regulator of uterine fibroid growth. In addition, AR trinucleotide repeat polymorphisms are associated with susceptibility to uterine fibroids. Hsieh et al. has found that the distribution of CAG repeats of the AR gene differs between patients with uterine fibroids and normal individuals [49]. Another study demonstrated that Asian Taiwanese women with the S genotype (27 CAG repeats) have a higher risk of developing uterine fibroids [49]. However, women with 20 CAG repeats have more possibility of developing uterine fibroids in Asian Indian women [50]. Inversely, AR gene (CAG)n-repeat polymorphism was not associated with the risk of uterine fibroids in Brazilian women [51]. Therefore, AR gene polymorphism is inconsistent among different ethnic groups.

The treatments of uterine fibroids mainly include surgery and drug treatment. Myomectomy can radically remove the lesions. However, surgery also causes damage to the uterus and disrupts fertility [52]. Medicines commonly used include GnRH agonists (GnRH-a), danazol, progesterone, mifepristone, and some traditional Chinese medicines [53]. The mechanisms by which danazol inhibits the growth of fibroids include increasing the metabolism of estrogen and progesterone and inhibiting the production of both hormones (Figure 2). In patients with uterine fibroids, danazol normalizes estrogen metabolism by reducing the expression of aromatase cytochrome P450 [54]. In particular, danazol affects the production of FSH and LH by inhibiting the release of GnRH and ultimately inhibits the production of estrogen and progesterone [55]. A clinical study found that the volume of uterine fibroids in women decreased after danazol treatment, and the reduction persisted for six months or even longer after the end of the treatment [56]. In addition, danazol can prolong the therapeutic effect of GnRH-a on uterine fibroids. A cohort study that enrolled 21 patients treated with 100 mg danazol for six months after GnRH-a treatment found that the rebound of uterine fibroids was about 30% less than that of the control group [57]. During GnRH-a treatment, bone mineral content of patients decreased significantly, whereas this situation improved during danazol treatment. Additionally, danazol has been shown to affect the hemodynamic effects of uterine fibroids, with a positive correlation between therapeutic effects and increased uterine artery impedance [58].

## 5. Androgen Signaling in Endometrial Polyps

Endometrial polyps are one of the most common types of endometrial lesions [59]. Endometrial polyps are single or multiple smooth masses covered by epithelium due to the overgrowth of localized stromal cells. It is estimated that 8–12% of women suffered from endometrial polyps during their reproductive years [60]. The growth of endometrial polyps is stimulated by estrogen and can cause varying degrees of endometrial hyperplasia, which may progress to malignant diseases. Hysteroscopic resection is considered necessary for the treatment of endometrial polyps [61]. Several risk factors associated with endometrial polyps include obesity, late menopause, and the use of tamoxifen [62,63]. The effect of hormones on polyp formation is not well understood. In premenopausal women, decreased levels of estrogen receptors and progesterone receptors in polyp stromal cells may make polyps less sensitive to cyclic hormonal changes [64]. In one study, 13 postmenopausal women with benign endometrial lesions, including endometrial polyps, had significantly higher serum DHEA, DHEAS, and T levels than normal postmenopausal women [65]. It has been shown that endometrial polyps express the aromatase p450 enzyme, which stimulates the conversion of free T into E2, thereby inducing the proliferation of polyps [66]. This may be the reason why endometrial polyps are insensitive to T. Among 258 postmenopausal women who received estradiol and testosterone implants for the relief of menopausal symptoms, endometrial hyperplasia and endometrial polyps were found to be more prevalent [67]. Therefore, the combination of progestin and estrogen is superior to the combination of androgen and estrogen for postmenopausal women.

## 6. Androgen Signaling in Endometrial Hyperplasia

**Figure 3:**
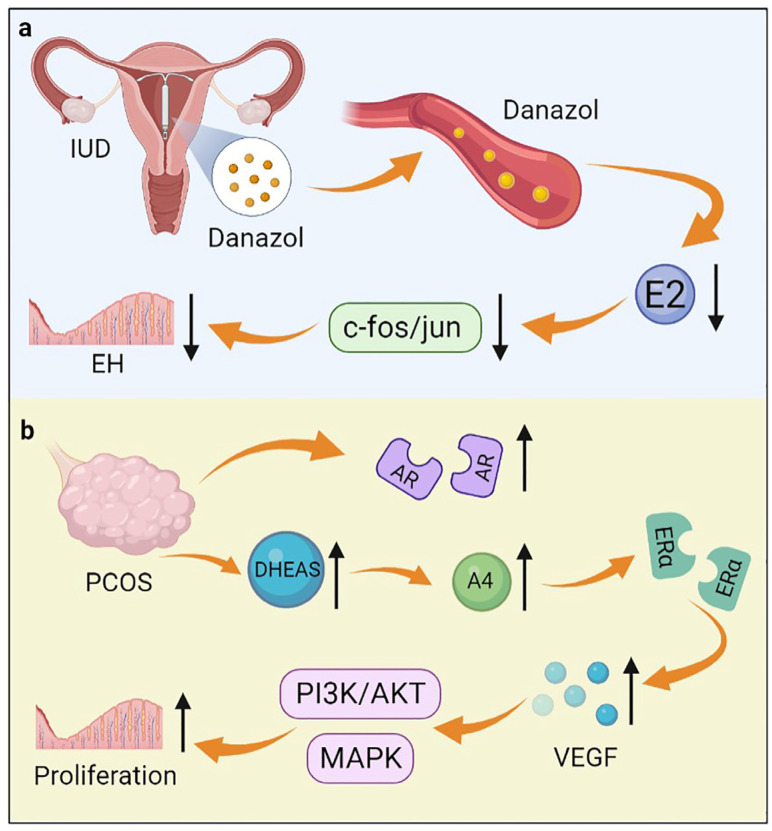
The role of androgen in endometrial hyperplasia. (**a**) An IUD containing danazol inhibits estrogen-induced c-fos/jun expression by releasing danazol, thereby inhibiting endometrial hyperplasia; (**b**) DHEAS and AR are increased in women with PCOS. DHEAS is converted to A4, which increases the expression of VEGF through ERα. VEGF further activates the MAPK and PI3K/AKT pathways, thereby promoting the occurrence of endometrial hyperplasia.

Endometrial hyperplasia is characterized by an increase in volume and structural changes in the endometrium, with a ratio of endometrial glands to mesenchyme greater than 1:1 [68]. EH results from chronic estrogen excess or progesterone deficiency [68]. There is strong evidence that EH increases the risk of concurrent endometrial cancer or endometrial cancer progression [69]. As defined by the World Health Organization (WHO) in 2014, EH includes hyperplasia with and without atypia [70]. Androgen plays an important role in the development of EH. The concentration of androgen in the peripheral blood of premenopausal women with EH was significantly higher than that of normal women [71]. Ito et al. found that the role of androgens in EH may be primarily regulated by serum T rather than DHT because of the lack of 5α-reductase [72]. Conservative medications for EH include progestins, GnRH-a, and danazol [73]. It is reported that danazol released from the danazol-releasing intrauterine device (D-IUD) has a direct therapeutic effect on EH through local diffusion and blood transmission (Figure 3) [73]. The mechanisms of action of danazol on EH include inhibition of endometrial cell proliferation and reduction of estrogen synthesis [74]. After treatment with danazol, pseudodecidual changes occurred. Endometrial glands atrophied, and further, they were transformed into normal secretory endometrium, thus improving the symptoms of patients with EH [73]. An animal experiment demonstrated that danazol significantly reduced the incidence of atypical EH by inhibiting estrogen-induced c-fos/jun expression [75].

Polycystic ovary syndrome (PCOS) is a common endocrine disorder that affects 5–8% of women of reproductive age. PCOS is characterized by polycystic ovaries, elevated androgen levels, and menstrual irregularities [76]. There is a high prevalence of endometrial lesions such as endometrial hyperplasia, endometrial cancer, and embryo implantation failure in women with PCOS [77]. Prenatal hyperandrogenization (PH) is considered to be one of the main factors in the development of PCOS. Ferreira et al. found that PH caused an increase in the total thickness of the uterus and disturbed the cell cycle, which led to the development of EH [78]. About 20–30% of PCOS patients have excess androgens in circulation, especially DHEAS [79]. In women with PCOS, DHEAS is converted to high levels of A4 by steroidogenic enzymes. Then, A4 acts through ERα to increase the expression of vascular endothelial growth factor (VEGF), which activates the MAPK and PI3K/AKT pathways in an autocrine/paracrine manner, thereby promoting the development of EH (Figure 3) [80]. In addition, AR expression increased in the endometrium during the development of EH in women with PCOS [81]. It is confirmed that androgens induce the development of EH in PCOS patients through AR-mediated AMPK (Adenosine 5’-monophosphate (AMP)-activated protein kinase) -α activation [82]. In addition, Li et al. reported that the combination therapy of Diane-35 and metformin could treat EH and early-stage endometrial cancer in women with PCOS [83]. The exact mechanism is not clear but may be caused by the anti-androgenic properties of Diane-35 and inhibition of endometrial AR signaling.

## 7. Androgen Signaling in Endometrial Cancer

### 7.1. Dual Effects of Androgens

**Figure 4:**
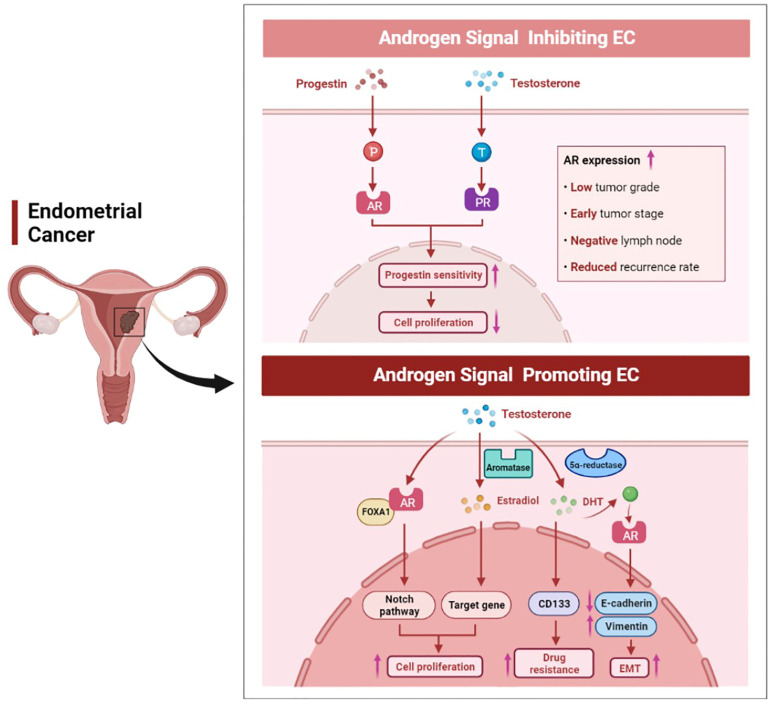
The dual role of androgen signaling in endometrial cancer. High AR expression was positively correlated with a lower grade, early stage, negative lymph node, and lower recurrence rate in EC. Progestin can inhibit tumor growth through AR signaling, and androgen can also bind to PR, thereby enhancing the progestin sensitivity of EC. On the other hand, androgen can promote EC cell proliferation, enhance EMT, and affect the resistance of EC cells to cisplatin.

Endometrial cancer is one of the most common gynecological malignancies in women and is mainly classified into type I and type II [84]. Nearly 80% of patients have type I EC, which is mainly endometrioid adenocarcinoma and has a better prognosis [85]. Type II EC consists of serous, clear cell, carcinosarcoma, and undifferentiated carcinoma, as well as other rarer types [86]. Several studies have reported an association between elevated serum androgen levels and the risk of EC. In postmenopausal patients with EC, elevated levels of DHEA, DHEAS, A4, and T were found in patients with EC compared to the healthy population [87]. Tanaka et al. found that patients with endometrioid adenocarcinoma had an 8-fold higher DHT concentration in the tissue/serum ratio than normal controls [88]. A limitation of these studies is that they only considered patients’ hormone levels over a single period. In recent years, two random Mendelian studies genetically analyzed the hormone levels of over 12,000 EC patients over their lifetimes, and they found that free T was associated with EC side effects [89,90]. In terms of mechanism, a high level of androgens is converted into estrogens by aromatase, resulting in increased levels of estrogen in tissues and promoting tumor cell growth (Figure 4) [91]. Aromatase is expressed not only in cancer cells, but also in stromal cells [92]. High expression of aromatase in stromal cells is associated with poor prognosis, suggesting an interaction between the tumor cells and stromal cells [93]. Qiu et al. found that AR bound to forkhead box A1 (FOXA1) and activated the Notch signaling pathway, thereby promoting EC cell proliferation [94]. Besides, androgens and AR have been shown to enhance cancer cell migration, epithelial–mesenchymal transition (EMT), and the number of EC stem cells [95]. Androgen can also affect the drug resistance of EC cells to cisplatin chemotherapy by increasing the expression of CD133 [95].

However, the effects of androgens on EC cells have inconsistent results compared to studies that directly measure the risk of androgens on EC. In vitro studies have shown that androgens, particularly A4, can inhibit the growth of endometrial epithelial cells [96]. It has been shown that the levels of androgens decrease with age in women, while the incidence of EC increases simultaneously [97]. Based on the action of aromatase, the decrease in androgen levels directly causes a corresponding decrease in the levels of estrogen in menopausal women. However, a decrease in estrogen levels does not reduce the incidence of EC. This may be because when androgen levels decrease, the inhibitory effect of androgen on EC cell proliferation is weakened [97]. This is consistent with the findings that androgen can inhibit the proliferation of Ishikawa cells, which are well-differentiated EC cells [98]. In addition, there is a correlation between an androgen signaling pathway and a progestin signaling pathway. It has been demonstrated that androgens increase PR expression in EC, thus exogenous androgen therapy may be an innovative therapy for EC patients who are insensitive to progestin treatment (Figure 4) [99]. What is more, progestin can reduce the stimulation of estrogen signaling and inhibit the proliferation of EC cells via upregulating the expression of AR [100,101]. It has been reported that MFE-296 endometrial cancer cells express AR in vitro, and both progestin and DHT treatment can inhibit the proliferation of MFE-296 cells [102]. In mammalian cells expressing exogenous or endogenous AR, medroxyprogesterone acetate (MPA) exerts a pronounced androgen agonistic effect. A significant increase in AR transcriptional activity was observed in the COS-1 cell line after MPA treatment in vitro [103]. In conclusion, due to hormone interaction, cell specificity, androgen type, androgen exposure time and other uncertain factors, it may be difficult to determine the role of androgen in EC [104].

### 7.2. AR Expression in EC

AR signaling in EC has both oncogenic and tumor suppressive effects, which may depend on the different stages of EC (Figure 4). In a mouse model of type I EC, a third-generation AR antagonist, enzalutamide inhibited EC cell proliferation and increased cancer cell apoptosis in a dose-dependent manner. However, enzalutamide increased tumor invasion and metastasis in advanced tumors [105]. A number of studies showed that AR expression was significantly increased in type I EC and was associated with favorable prognostic factors such as early stage of cancer, low grade, lymph node negativity, and reduced tumor recurrence rate [106,107,108]. Besides, decreased AR expression has been reported to lead to methylation of the MLH1 gene, which, in turn, causes mismatch repair (MMR) deficiency and promotes endometrial carcinogenesis [109]. Taken together, these data suggest that AR may play different roles in the development of EC. AR expression levels can also be used to predict the occurrence of EC. The N-terminal domain of the human AR gene contains CAG repeats that affect transcriptional efficiency, and the length of the CAG sequence is inversely related to AR activity [110]. It was shown that the CAG sequence was longer in patients with EC than in normal people, thus resulting in reduced AR activity. The decrease of AR activity reduced the anti-proliferative effect on endometrial cells and promoted endometrial cell carcinogenesis [110]. Thus, the susceptibility of an individual to EC can be predicted based on the length of the CAG repeat sequence in the AR gene. Furthermore, hypermethylation of CpG islands on AR genes leads to AR inactivation and is associated with the development of stage III and IV EC [111].

Previous studies have focused on endometrioid adenocarcinoma, with fewer studies on high-grade EC. Undifferentiated endometrial cancer (UC) and dedifferentiated endometrial cancer (DEAC) are high-grade endometrial cancers with a lower incidence and a greater capacity for invasion and metastasis [112]. AR is reported to be highly expressed in UC/DEAC (63%), serous carcinomas (88%), and carcinosarcomas (80%) [113]. In addition, AR is positive in these high-grade endometrial cancers lacking ER or PR expression [113]. These findings suggest a potential therapeutic role for androgen inhibitors in the treatment of patients with these tumors. In prostate cancer and AR-positive triple-negative breast cancer, androgen antagonists have shown good therapeutic effects [114,115]. AR immunostaining allows screening of a group of AR-positive EC that would benefit from anti-androgen therapy. Therefore, androgen antagonists are novel therapeutic agents for high-grade non-endometrioid cancers that currently lack effective endocrine therapy [116].

## 8. Androgen Signaling in Infertility Associated with Endometrial Dysfunction

Androgens have a beneficial effect on the regulation of pregnancy. It is reported that androgens regulate endometrial decidualization and embryo implantation [117]. Endometrial decidualization is the proliferation and differentiation of endometrial mesenchymal cells, which occurs during pregnancy [118]. Blocking androgen signaling leads to a significant reduction in the expression of key indicators of decidualization such as PRL and IGFBP1 [119]. In human embryonic stem cells, the expression and activity of the androgen synthesis-related enzymes AKR1C3 and SRD5A1 are associated with decidualization in vitro, suggesting that these enzymes may be targets for the treatment of infertility associated with endometrial dysfunction [119]. Androgens also regulate the expression of endometrial receptivity-related markers in human embryonic stem cells [119]. Gibson et al. reported that supplementation with DHEA could improve the decidualization of the endometrium and promote the initiation of pregnancy [120]. Interestingly, treatment with supraphysiological levels of DHEA is not effective and decidualization is weakened [121]. Therefore, the therapeutic effect of DHEA may be dose-dependent, and it is suggested to use a wider therapeutic window for treatment. Additionally, in mouse models, androgen deficiency delays embryo implantation, while excess androgens result in abnormal gene expression at the site of implantation [122].

Low androgen levels have been reported to be associated with decreased ovarian reserve (DOR) and lower pregnancy rates [123]. Supplementation with androgens or androgen precursors may improve ovarian function, thereby increasing embryo fertilization rates. In 89 patients with DOR treated with DHEA before in vitro fertilization (IVF), clinical pregnancy rates were significantly increased [124]. However, in a study of DHEA supplementation in women with DOR undergoing IVF or intracytoplasmic single sperm injection (ICSI), a higher pregnancy rate was found in the control group than in the DHEA-treated women [125]. Although DHEA supplementation in these trials was intended to support ovarian function, DHEA did not appear to improve ovarian function in terms of ovulation rates or the number of embryos fertilized [124]. Moreover, the group supplemented with DHEA had a lower miscarriage rate, and almost half of the pregnancies occurred before IVF [124]. It is more likely that the ability of DHEA to increase pregnancy rates is due to the improved condition of endometrium. Therefore, androgens can be used in assisted reproduction techniques to improve pregnancy outcomes of patients, but more research is needed to investigate the effects of androgens on ovary andendometrium. However, many women are intolerant to the side effects produced by androgens. The selective androgen receptor modulator (SARM) and new enzyme selective modulator such as AKR1C3 provide a more targeted treatment to reduce the side effects [117]. Targeted androgen therapy can also be performed through intrauterine devices to provide an effective treatment for improving pregnancy outcomes.

## 9. Conclusions

Androgen signaling is involved in many uterine physiological processes, and there are varying degrees of evidence for its role in benign uterine lesions and malignant progression. Androgens have been shown to be effective in many uterine diseases; however, the side effects of androgens have limited their use in women’s diseases. SARM is a new class of compounds that exhibit agonist and antagonist effects on AR in target tissues with good efficacy and few side effects [126]. SARMs have been used to treat breast cancer, but there have been no clinical trials for endometrial cancer, endometriosis, and other uterine-related diseases, so further research is needed [127]. A growing number of epidemiological and clinical trials support the further exploration of AR-targeted drugs in a variety of uterine-related diseases (Table 1). The next generation of AR-targeted drugs will play a broad therapeutic role in uterine-related diseases in the coming decades.

## Figures and Tables

**Table 1:** Ongoing studies testing androgen-related therapies in uterine diseases.

Conditions	Study Phase	Sample Size	Interventions	NCT Number
Endometrial Cancer	II	56	Drug: danazol	NCT00003946
Endometrioid Endometrial Cancer	II	69	Drug: Enzalutamide Drug: Carboplatin Drug: Paclitaxel	NCT02684227
Endometriosis	II	30	Drug: Vaginal Danazol Drug: Oral Danatrol	NCT03352076
Endometriosis Ovarian Cysts Infertility	IV	150	Drug: Danazol	NCT01779232
Endometriosis	II	66	Drug: Danazol Once Weekly Drug: Danazol Twice Weekly	NCT00758953
Hypoactive Sexual Desire Disorder	III	1271	Drug: Testosterone Transdermal System	NCT00467259
Postmenopausal Women	III	32	Drug: tibolone Drug: Tibolone 2.5 mg Drug: CE/MPA	NCT00745108
Postmenopause Osteoporosis	IV	35	Drug: Tibolone Drug: Estradiol Drug: Estradiol + MPA	NCT00294463

## Data Availability

No new data were created or analyzed in this study. Data sharing is not applicable to this manuscript.
